# The State Educational Systems

**Published:** 1903-04

**Authors:** 


A Monthly Review of Medicine and Surgery.

*EDITOR:*

WILLIAM WARREN POTTER, M. D.

All communications, whether of a literary or business nature, books for review and exchanges should be addressed to the editor: 284 Franklin Street, Buffalo, N. Y.

Vol. XLII.—LVIII. APRIL, 1903. No. 9.

### The State Educational Systems.

THE friction between the two heads of the educational departments of the state has been so long the subject of discussion in educational circles, in the newspapers, and among the people, that it ought by this time to be pretty well understood. It is difficult to comprehend why the department of public instruction was ever created; why the common schools were committed to the charge of a superintendent whose tenure is political; why the administration of the common schools was separated from that of the higher educational institutions. This, we say, is difficult to understand when the University of the State of New York, in existence since 1784, has stood ready to administer the entire educational system in a faithful, non-partisan, economic, and thoroughgoing manner.

Whatever reasons may have been considered adequate for establishing the department of public instruction have now ceased to be cogent for its continuance, in view of the fact that it seeks to control matters clearly within the province of the regents, and that it even sets itself over and above that body in many respects. It is quite time that this misfit between these two systems which has well-nigh become a scandal, should be corrected, that the intolerable situation now existing should be brought to an end. It should be done, too, in a permanent fashion in order that it may not be possible to witness its renewal.

Several methods of accomplishing this result have been proposed, but of all that of Senator Stevens, of Attica, representing the 46th senatorial district, appears the most feasible and efficient. The text of the bill is as follows:

The offices of state superintendent of public instruction and of the deputies of such superintendent are hereby abolished, and the powers, functions and duties of such offices are hereby continued and vested in the University of the State of New York and shall hereafter be exercised and performed by its regents, or, as they shall direct, by their officers and appointees and the persons, other than such deputies, now in office as appointees of the present superintendent; and his said appointees shall, subject to the direction and during the pleasure of the said regents, continue in their present positions and receive their present compensation. The present state superintendent of public instruction and his deputies shall be entitled to receive their present salaries until the expiration of the term for which the said superintendent was elected, but they shall hereafter have, exercise and perform only such powers and duties as the said regents shall expressly direct.

The superintendent is elected by the legislature for three years; the regents are appointed for life; the former is a political officer; the board of regents is a constitutional body, non-political in character. If the foregoing bill is enacted into law the educational interests of the state will be removed from the political arena,—a much to be desired consummation and one which every true friend of education will approve.

The medical profession takes great interest in the cause of education, and especially in the university and its administration as it exercises control over professional education in the state, and we may add with entire truthfulness, to the entire satisfaction of the colleges, profession, and people in general. The Medical Society of the County of Erie held a special meeting, March 18, 1903, at which the following preamble and resolution were unanimously adopted:

WHEREAS, The Medical Society of the County of Erie, State of New York, has called this meeting of members of the medical profession of the City of Buffalo and County of Erie; and

WHEREAS, As physicians and representatives of our several societies, we are unreservedly and unequivocally committed to the right of individual opinion and also to the paramount necessity for unity in action; and

WHEREAS, We have for years noted with regret the lack of unity in the educational system of our state; and,

WHEREAS, Legislation is now under consideration for the purpose of bringing about a much needed unification; and

WHEREAS, We believe that education is the foundation of the protection and promotion of the public health, the safeguard of civil and religious liberty, the bulwark of civilisation, and that in education only the most intelligent methods should prevail, also that in education there should be unity of direction; and

WHEREAS, The University of the State of New York was conceived in the highest wisdom and for more than a century has been conducted with marvelous ability and success, and now holds the universal confidence of the educators and the educated; and

WHEREAS, It is the purpose of a bill now before the legislature at Albany, known as the Stevens bill, to provide for such unification of the educational system of the state under the direction of the regents of the university; therefore,

*Resolved,* That the medical profession of the City of Buffalo and County of Erie, hereby declares itself heartily in favor of the Stevens bill; that we urge our representatives to use all honorable efforts to secure its passage; that a copy of this declaration be sent to each member of our delegation and a copy to the Honorable James Russell Parsons, Jr., secretary of the university, regents' office, Albany, N.Y.

The foregoing is so expressive of the general feeling of the medical profession which is united on this important question, that we need not further extend the discussion in this place; but it is pertinent to urge every physician to exert his individual influence in favor of Senator Stevens's bill.

